# Roughness Factors of Electrodeposited Nanostructured Copper Foams

**DOI:** 10.3390/nano13233011

**Published:** 2023-11-23

**Authors:** Eduard E. Levin, Dmitriy A. Morozov, Vsevolod V. Frolov, Natalia A. Arkharova, Dmitry N. Khmelenin, Evgeny V. Antipov, Victoria A. Nikitina

**Affiliations:** 1 Department of Chemistry, Lomonosov Moscow State University, Moscow 119991, Russia; dmitrii.morozov@chemistry.msu.ru (D.A.M.); frolov_vsevolod@mail.ru (V.V.F.); antipov@inorg348-1.chem.msu.ru (E.V.A.); 2 Federal Scientific Research Centre “Crystallography and Photonics” of the Russian Academy of Sciences, Moscow 119333, Russia; natalya.arkharova@yandex.ru (N.A.A.); xorrunn@gmail.com (D.N.K.); 3 Center for Energy Science and Technology, Skolkovo Institute of Science and Technology, Moscow 121205, Russia

**Keywords:** Pb UPD, electrodeposition, real surface area, Cu foam

## Abstract

Copper-based electrocatalytic materials play a critical role in various electrocatalytic processes, including the electroreduction of carbon dioxide and nitrate. Three-dimensional nanostructured electrodes are particularly advantageous for electrocatalytic applications due to their large surface area, which facilitates charge transfer and mass transport. However, the real surface area (RSA) of electrocatalysts is a crucial parameter that is often overlooked in experimental studies of high-surface-area copper electrodes. In this study, we investigate the roughness factors of electrodeposited copper foams with varying thicknesses and morphologies, obtained using the hydrogen bubble dynamic template technique. Underpotential deposition (UPD) of metal adatoms is one of the most reliable methods for estimating the RSA of highly dispersed catalysts. We aim to illustrate the applicability of UPD of lead for the determination of the RSA of copper deposits with hierarchical porosity. To find the appropriate experimental conditions that allow for efficient minimization of the limitations related to the slow diffusion of lead ions in the pores of the material and background currents of the reduction of traces of oxygen, we explore the effect of lead ion concentration, stirring rate, scan rate, monolayer deposition time and solution pH on the accuracy of RSA estimates. Under the optimized measurement conditions, Pb UPD allowed to estimate roughness factors as high as 400 for 100 µm thick foams, which translates into a specific surface area of ~6 m^2^·g^−1^. The proposed measurement protocol may be further applied to estimate the RSA of copper deposits with similar or higher roughness.

## 1. Introduction

Nanostructured copper-based materials with a high surface area (such as oxide-derived copper [1,2,3,4,5,6,7], nanoporous copper films produced by electrochemical dealloying [8,9,10] and copper foams produced by hydrogen bubble templating [11,12,13,14]) are under intense investigation due to the prospects of such materials for application as catalysts for CO_2_ [1,2,3,4,5,6,7,11,12,13,14] and nitrate [1,15,16,17] reduction, which are two promising processes aimed at decarbonizing industry and reducing the environmental risks associated with climate changes due to accumulation of CO_2_ in the atmosphere. In particular, nanostructured copper foams are unique electrocatalysts, as their selectivity depends on surface roughness, specific hierarchical porous morphology and capture of reaction species in the pores [18]. Distinguishing the effects of a high surface area and reacting species’ confinement inside the pores on the activity of the catalyst is challenging for nanoporous electrocatalysts, as reliable estimates of real (RSA) or electrochemically active surface area (EASA) of the electrocatalyst are required [19]. RSA of catalytic coatings and particles is a crucial parameter, which directly affects the conclusions on the intrinsic activity of a given material [20,21].

For electrocatalytic materials obtained in the form of powders, the specific surface area is most frequently determined by physisorption measurements using the Brunauer–Emmett–Teller theory. Yet, such measurements become problematic when the surface area of electrodeposited catalysts is to be evaluated due to typically much lower loadings of active material and therefore low accuracy of techniques based on physisorption. Additionally, when the materials are fabricated into electrodes, the parameter of interest is the EASA, which refers to the actual surface available for the electrochemical reaction, and not the specific surface area [22]. The need for sample heating in order to obtain accurate measurements is another obstacle, as heating increases the surface diffusivities of metal atoms, which typically results in coarsening of nanostructured electrocatalysts and a corresponding decrease in the RSA. These shortcomings can be overcome by electrochemical methods [22].

Double-layer capacitance and the charge required to form a monolayer of metal at potentials more positive than the equilibrium potential (underpotential deposition, UPD) can be directly recalculated into the RSA [22]. Double-layer capacitance measurements for the estimation of the RSA of electrocatalysts are most commonly based on recording CVs in the double-layer region in electrolytes, where no faradaic reactions occur (an example for copper catalyst can be found in Ref. [2]). This procedure can hardly be recommended for rigorous determination of RSA of copper-based electrodes, since finding the true double-layer region on polycrystalline copper electrodes is not trivial, as specific adsorption of anions, hydroxyl species and organic impurities influence the capacitive currents greatly. Additionally, such CVs must be registered with an analog potentiostat [23,24], which may not be available in many laboratories. Capacitance values may be obtained from electrochemical impedance spectroscopy data. However, for porous electrodes rather complex equivalent circuit modeling should be performed in order to extract physically meaningful estimates from the experimental data, while fitting to simple circuits with constant phase elements may result in unrealistic values of roughness factor R_f_ [25,26,27] (the ratio between the measured surface area of and the corresponding geometric area). Recently, a method based on analyzing cyclic voltammograms of porous copper materials registered in alkaline media was proposed to evaluate RSA [28,29]. The analytical signal was attributed to the peaks of copper oxidation with the formation of the corresponding oxides (not monolayers). However, this method seems only applicable to materials with similar morphologies but different geometric surface areas, as the kinetics and thus the heights of the oxidation peaks are expected to vary greatly for different sizes of primary particles in copper-based materials. Surface area quantification using the methodology based on the UPD of metals is therefore the most attractive option in terms of reliability of the estimates and transparency of the data treatment to obtain R_f_. Approaches for RSA determination based on UPD of copper were shown to be particularly useful for assessing the surface area of nanoporous gold produced by electrochemical dealloying [25,30].

The RSA of noble metal electrocatalysts can be reliably determined through the UPD of hydrogen, Cu, Pb, and Tl adatoms, as well as CO stripping [22,31,32]. For copper, information on the RSA can be obtained through UPD of lead or thallium, with the former being more frequently applied due to lower toxicity [33,34,35,36,37,38]. However, studies relevant to the application of Pb UPD to assess the RSA of highly dispersed copper-based materials with complex morphologies are relatively scarce. Most of the reported Pb UPD studies use single-crystal copper electrodes, which typically exhibit negligible surface roughness [24,33,36,38]. Only a few studies aimed to evaluate the RSA of polycrystalline copper electrodes by UPD of Pb. The RSA of a polycrystalline copper electrode with a roughness factor R_f_ of 1.15 was evaluated in Ref. [34], while in Ref. [35], Pb UPD was applied to estimate the RSA of a copper material, produced by repeated oxidation reduction in polycrystalline copper, resulting in an R_f_ of 1.8. Roughness factors of 20–30 were obtained by Pb UPD measurements of reduced copper oxide [19] and nanoporous Cu film produced by electrochemical dealloying [8].

For micron-sized copper powders with a low specific area of about 0.5 m^2^·g^−1^, the agreement between the RSA values obtained from double-layer, UPD of adatoms and microscopy assessment was found to be satisfactory [39]. However, for the materials with hierarchical porosity, special care should be taken to ensure the participation of the entire surface area in the process of double-layer charging or monolayer formation, with the diffusion limitations in the pores provoking underestimation of the RSA. A recent report on the R_f_ determination for a copper foam electrode emphasized the inapplicability of the UPD of Pb for the quantification of the RSA of copper foam, as the R_f_ values derived from double-layer measurements were an order of magnitude higher than those obtained via the UPD of Pb [26]. Such discrepancies are not surprising, as the process of monolayer Pb formation is complex and depends on the pH, electrode pretreatment, and the nature of the anions in the electrolyte [24,33,38]. Moreover, the process is electrochemically irreversible as a surface phase transition occurs in the course of monolayer formation [36,40]. Due to the slow kinetics of the Pb UPD process and additional complications arising from diffusion limitations in the pores of nanostructured materials, a systematic and comprehensive study on the kinetics and limitations of the Pb UPD process in porous nanostructured copper electrodes is needed.

The present work is aimed at assessing the roughness factors of copper foams with different thicknesses and morphologies (R_f_ about 200 and 400) and illustrating the applicability of the Pb UPD method under the optimized conditions for reliable estimation of RSA for highly dispersed copper-based electrocatalysts.

## 2. Materials and Methods

### 2.1. Electrodeposition

Copper foam samples were obtained by electrodeposition in a two-electrode configuration under a constant current mode. Two solutions were used for electrodeposition: (1) 0.075 M CuSO_4_ + 0.5 M H_2_SO_4_ (Cu_f_ samples); (2) 0.075 M CuSO_4_ + 0.5 M H_2_SO_4_ + 0.05 M KCl (Cu_f,Cl_ samples). The solution compositions were based on the data reported in Refs. [41,42], and the Cl^−^ ions were added to obtain the deposits with smaller primary particles, based on the results from Ref. [41]. The electrodeposition was carried out in a rectangular glass vessel. A copper foil of 99.96% purity (thickness of 0.25 mm and an area of 1 cm^2^) was used as the working electrode, and a copper foil of 99.99% purity (thickness of 1 mm and an area of 1.5–2 cm^2^) was used as the sacrificial anode. The distance between the electrodes was fixed to 2 cm. Prior to electrodeposition, the working electrode surface was pretreated as follows: etched in hot H_2_SO_4_ solution (195 g·L^−1^, 60 °C) until the oxide film was removed (usually about 10–15 s), washed with water, dried and activated in another H_2_SO_4_ solution (17.5 g·L^−1^, room temperature) for 1–3 s. Electrodeposition was performed at a current of 3 A for 10–30 s at room temperature. After deposition, the samples were rinsed with 300–350 mL of deionized water using a water mist system and dried in a vacuum oven (70 °C, 0.07 bar) for 30 min. Current efficiencies for Cu_f_ and Cu_f,Cl_ deposition were 21 ± 2% and 22 ± 2%, respectively. The total volume of pores was 92 ± 1% for both series of specimens. The details on the determination of both parameters are provided in Section S1 of the Supporting Information.

### 2.2. S Copper Foam Characterization

X-ray diffraction (XRD) patterns of the foams were collected using a Malvern Panalytical Aeris diffractometer (Bragg–Brentano geometry, CuKα radiation, PIXcel^3D^ detector). Copper particle suspensions were prepared using an ultrasonic bath, in which a foam-coated foil was placed in a glass beaker containing isopropanol. The suspension was then drop cast onto a single-crystal silicon wafer (zero-background holder) for XRD pattern collection. Scanning electron microscopy (SEM) images were obtained using an FEI Scios (Schottky field emission gun, Everhart–Thornley detector (positive bias), landing energy 2–5 kV) scanning electron microscope. The particle size distribution and pore size distribution were calculated by image analysis in the Fiji software v. 1.54f [43]. To determine the particle size distribution, a grain count was used, in which the projected area of individual grains was determined and recalculated to the diameter of particles with the same area (571 for Cu_f_ and 186 for Cu_f,Cl_). The pore size distribution was estimated by granulometry by closing with a disk structuring element [44]. Cross-sections were prepared by cutting foams on Leica EM TXP target preparation device followed by Ar^+^ ion milling using Hitachi IM4000Plus ion milling system (acceleration voltage 5 kV, ion current 1 mA). Transmission electron microscopy (TEM) images were taken in bright-field scanning TEM (BF STEM) mode on a FEI Tecnai Osiris transmission electron microscope operating at 200 kV. TEM samples were prepared by drop casting a suspension of foam particles in isopropyl alcohol onto a Cu lacey carbon grid.

### 2.3. Electrochemical Measurements

Prior to the UPD measurements, the Cu_f_ and Cu_f,Cl_ samples were pre-reduced in a solution of 0.1 M KOH by sweeping the potential from the open circuit value to −1.1 V vs. HgO/Hg (1 M NaOH) reference electrode and then holding this potential for 5 min. During this time the current reached a stationary value.

Electropolished copper foil was used for a number of UPD experiments. For this purpose, the foil was degreased with isopropyl alcohol followed by electrochemical degreasing in solution of 25 g·L^−1^ Na_3_PO_4_ + 25 g·L^−1^ Na_2_CO_3_ at 60 °C with a current of 0.1 A cm^−2^ for 4 min. After electrochemical degreasing, the foil was immersed in distilled water at 60 °C until complete surface wetting was achieved (typically 1–2 min). The foil was washed with distilled water and electropolished in 10 M H_3_PO_4_ aqueous solution for 30 s at 5 V. After electropolishing, the foil was soaked in 0.2 M HCl for 30 min, then washed with water and dried with compressed air.

Pb UPD measurements were performed in an electrochemical cell with copper foam as a working electrode, graphite rod as a counter electrode, and 3 M AgCl/Ag as a reference electrode. The working electrode (ca. 15 mL) and counter electrode compartments were separated by a porous glass frit. The working electrode solution was de-aerated prior to the measurements for 30 min, and argon flow was maintained above the solution during the measurements. Special care was taken to avoid the contact of the copper foam electrode with the aerated solution at open circuit potential. To minimize diffusion limitations in the pores of the foams, in most of the UPD measurements (both voltammetric and chronoamperometric), the solution in the working electrode compartment was stirred using a magnetic stirrer at a rate of 100–400 rpm.

Perchloric acid (puriss. p.a., Sigma Aldrich, St. Louis, MO, USA), NaClO_4_ (>99.9%, Sigma Aldrich, USA), PbO (>99.5%, Lenreaktiv, St. Petersburg, Russia), HCl (puriss, p.a., Merck, Darmstadt, Germany), and KCl (>99.9%, Component-reaktiv, Moscow, Russia) were used to prepare the Pb UPD solutions. A constant perchlorate concentration of 0.1 M was maintained, and the pH of the solution was adjusted to 1, 2, 3 and 4 potentiometrically. The UPD charge was determined by integrating and then averaging the anodic and cathodic branches of the voltammograms after subtracting the background response in a 0.1 M NaClO_4_ + 1 mM HCl solution, which was determined in the potential region before the onset of hydrogen evolution. The solution was de-aerated with argon for 30 min before starting the measurements. The specific charge value was assumed to be 310 µC·cm^−2^, which is the value of a closely packed Pb monolayer [36,40].

All the electrochemical measurements were performed using Biologic SP-50 and Autolab 302N potentiostats.

## 3. Results and Discussion

### 3.1. Structure and Morphology of Copper Foams

Copper electrodeposition at high current densities was used to obtain porous deposits with different surface areas [42]. Such a deposition regime is a competitive process between metal deposition and hydrogen evolution, which acts like a dynamic template. Rapidly growing metal forms a foam whose walls are made up of dendrites. There are two sources of the hierarchical porosity characteristic of such metal deposits: (1) hydrogen bubbles growing during gas evolution, and (2) space-filling by dendrites within the walls. The first source is largely dependent on the solution properties, while the second is controlled by the particle morphology. Two types of copper foam samples were obtained: (1) from the solution 0.075 M CuSO_4_ + 0.5 M H_2_SO_4_ (Cu_f_) and (2) from the same solution with the addition of 0.05 KCl (Cu_f,Cl_). The addition of chloride was previously shown to have a catalytic effect on both underpotential and overpotential deposition of copper, presumably due to the changes in the solvation shell structure of Cu^2+^ in the presence of large anions [45]. Faster deposition kinetics results in smaller primary particles in the deposit [41], which allowed us to obtain high-surface-area samples with different R_f_ values.

**Figure 1:**
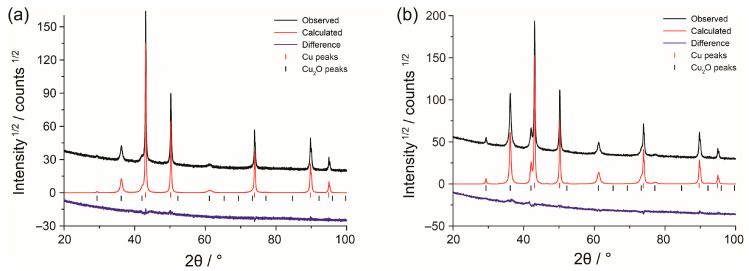
XRD patterns of the Cu_f_ (**a**) and Cu_f,Cl_ (**b**) samples.

XRD patterns of foam specimens are shown in Figure 1. In addition to the peaks of metallic copper, there are other peaks that belong to cupric oxide Cu_2_O, which forms due to oxidation in air. The weight fraction of Cu_2_O, determined by full-profile analysis, is higher for Cu_f,Cl_ foam (36.7 ± 0.5%) than for Cu_f_ foam (14.0 ± 0.5%).

**Figure 2:**
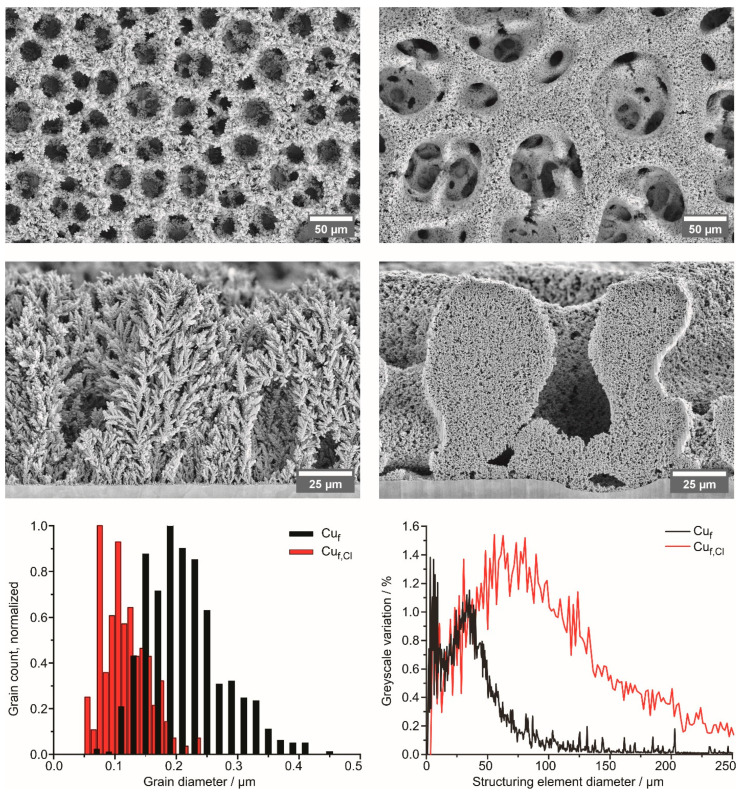
SEM images of the Cu_f_ (**top left**) and Cu_f,Cl_ (**top right**) foams. Cross section images of the Cu_f_ (**middle left**) and Cu_f,Cl_ (**middle right**) foams. Grain size distributions (**bottom left**) and granulometry curves (**bottom right**).

The morphology of the deposits is shown in Figure 2. The difference in the structure of foams is both in the shape of the constituent grains and in the pore structure. The Cu_f_ specimen shows a distinctive dendritic structure with a six-fold axis of symmetry along a dendrite growth direction (Supplementary Materials Figure S1). The addition of 0.05 M KCl strongly influences the pore structure (Figure 2) and prevents the formation of dendrites (Supplementary Materials Figure S1). Trace amounts of Cl^–^ were observed in the electron-probe-induced X-ray emission spectra during scanning electron microscopy examination. At the same time, no K emission lines were detected. Therefore, we assume that all the Cl- belongs to minute amounts of CuCl formed together with Cu during deposition. Being suspended, the deposited foam fragments retain their structure, as seen on their HAADF STEM images (Supplementary Materials Figure S2). The same features can be observed in the images of cross-sections of the deposits (Figure 2). While the Cu_f_ specimen is dendritic, the addition of KCl resulted in a sponge-like structure. For both foams, macroscopic pores extend through the whole deposit down to the substrate. Grain sizes, corresponding to maxima of grain size distribution (Figure 2), differ by a factor of two: 195 nm for the Cu_f_ and 107 nm for Cu_f,Cl_. This is consistent with the higher Cu_2_O content in the Cu_f,Cl_ sample, as more surface area is exposed to air, resulting in a higher oxide content not connected with the presence of chloride. The difference in pore structure, as determined using granulometric curves (Figure 2), is the following. Both curves have two broad maxima, the first of which (below 12 μm) corresponds to voids in the pore walls, and the second to the pores themselves. The second maximum for the Cu_f_ is 33 μm, and for the Cu_f,Cl_ is 70 μm.

### 3.2. Pb UPD

At the first stage, voltammetric experiments on the adsorption/desorption of lead adatoms (Supplementary Materials Figure S3) were carried out on copper foil with a low roughness factor R_f_ (electropolished foil according to the procedure described in the Experimental Section). The solutions used for the UPD studies were 0.1 M HClO_4_ + 1 mM Pb(ClO_4_)_2_ and 0.1 M HClO_4_ + 1 mM Pb(ClO_4_)_2_ + 1 mM HCl, as previous studies indicate that Cl^−^ ions accelerate Pb adatoms deposition/stripping kinetics [24,33,38]. Supplementary Materials Figure S3 shows a typical irreversible cyclic voltammogram (CV) for Pb underpotential deposition/stripping on polycrystalline copper foil. In agreement with the literature data, a small (10–30 mV) shift of the current maxima towards more negative potentials is observed when Cl^−^ is added to the solution [33,38], while the plating and stripping peaks narrow as a result of faster kinetics at higher overpotentials. The roughness factor for the polycrystalline foil calculated using the specific charge value of 310 μC·cm^−2^ is 1.2, which agrees with the previous estimates of roughness of the electropolished copper [34]).

#### 3.2.1. Pb^2+^ Concentration

**Figure 3:**
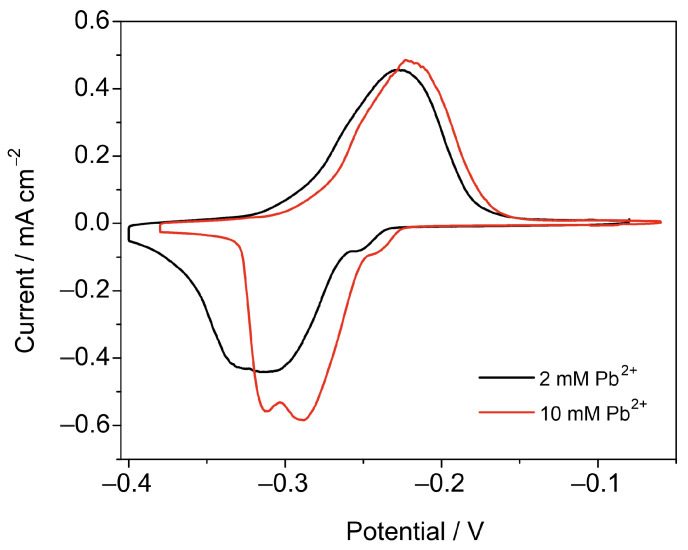
CVs of two Cu_f,Cl_ foams in solutions 0.1 M HClO_4_ + 1 mM HCl with 2 and 10 mM Pb(ClO_4_)_2_. Scan rate is 0.5 mV·s^−1^, with magnetic stirring.

To optimize the conditions for Pb UPD measurements on the samples with a high surface area, the effect of Pb^2+^ concentration on the shape of the CVs was studied. The CV in a more concentrated solution Figure 3 shows the CVs of a Cu_f,Cl_ sample (electrodeposition conditions: 3 A, 30 s) registered at 0.5 mV s^−1^ in solutions 0.1 M HClO_4_ + 1 mM HCl with 2 and 10 mM Pb(ClO_4_)_2_ added have narrower peaks at a slightly more positive formal potential. The R_f_ values calculated for the two Cu_f,Cl_ foams prepared under identical conditions were 254 (foam in solution with 2 mM Pb^2+^) and 246 (foam in solution with 10 mM Pb^2+^). Despite the difference in the R_f_ values between the two samples being less than 5%, a higher Pb^2+^ concentration in the UPD solution should be preferred for high-surface-area samples, as it increases the rate of Pb monolayer formation, as follows from the lower width of the deposition and stripping peaks.

#### 3.2.2. Effect of pH

In the next step, we focused on choosing an optimal pH value for the Pb UPD measurements on copper foams. The pH of the solution affects the kinetics of Pb UPD [36], the rate of HER at potentials below 0 V vs. RHE (which is low on copper and especially at Pb covered Cu, but HER is still thermodynamically possible at Pb UPD potentials [46]), as well as the rate for the dissolution of the residual amounts of copper oxides at the surface of the foams. The pH value should not affect the rate of oxygen reduction reaction, which is particularly important in Pb UPD measurements, as diffusion-limited reduction in the residual amounts of oxygen in the Pb UPD solutions contributes preferentially to the observed cathodic background currents during Pb monolayer formation [47]. However, local alkalinization [48] of the near-electrode region due to a constant supply of OH^−^ produced during oxygen reduction may alter the nature of the oxygen-containing adsorbates and the reversibility of the UPD process [36].

**Figure 4:**
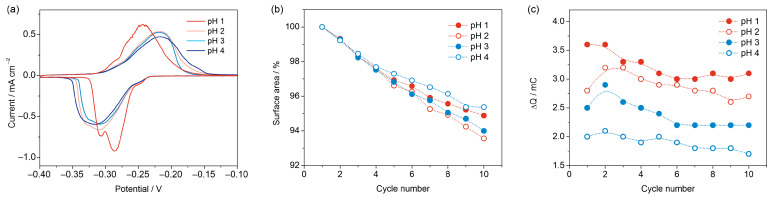
CVs of four Cu_f,Cl_ samples registered in Pb UPD solutions with pH 1, 2, 3 and 4 at 0.5 mV·s^−1^ scan rate, with magnetic stirring (**a**). Decrease in the surface area upon the subsequent cycling of the Cu_f,Cl_ samples in solutions with different pH values (**b**). The differences between the cathodic and anodic charges (ΔQ) at different pH (**c**).

Figure 4a shows the CVs of four Cu_f,Cl_ samples (3 A, 30 s), which were recorded in Pb UPD solutions with different pH values: 1, 2, 3 and 4 in the presence of 1 mM of Cl^−^. The Pb UPD kinetics are significantly faster in a solution with pH 1, whereas the peak shapes are similar at pH values between 2 and 4. These results contradict those obtained in Ref. [36], where the reversibility of Pb^2+^ UPD was worse at lower pH values compared to pH values of 1.5 and 3.5. This discrepancy may be explained by the presence of Cl^−^ ions in the UPD solution used in this study, which hinder the participation of oxygen species in site-exchange reactions during the UPD process [24,36,40].

An important aspect concerns the stability of high-surface-area copper deposits during cycling. To prevent changes in the surface area of copper-based electrodes due to the dissolution of copper oxides, all the samples should be pre-reduced before the UPD measurements. However, copper oxides may form when the samples are transferred from an alkaline solution after oxide reduction into the Pb UPD solution. This can occur due to oxidation in air or while contacting the UPD solution at the open circuit potential, as a result of copper corrosion in an oxygen-rich environment. We found that during cycling in a de-aerated Pb UPD solution, the surface area of the foam is slowly decreasing (Figure 4b). After 10 cycles at 0.5 mV·s^−1^ in the UPD solution, the surface area of the Cu_f,Cl_ foams drops by 5–7%; however, the changes do not show a systematic dependence on the pH. Furthermore, a comparable reduction in the Pb UPD charge is observed when holding the electrode at the open circuit potential. Thus, we conclude that the decrease in surface area is due to copper corrosion, with residual oxygen acting as the depolarizer [47].

In order to quantify the effect of pH on the magnitude of background currents at low scan rates, the cathodic and anodic charges were calculated from the CV, and the differences between these charges, which reflect the contribution of side reactions to the cathodic charge, are plotted vs. the cycle number in Figure 4c. As anticipated, a rise in pH results in a corresponding decrease in charge imbalance. While the charge arising from side reactions may be factored into the R_f_ calculation, a significant discrepancy between anodic and cathodic charges can compromise the precision of the RSA determination. Therefore, pH values 3 or 4 are preferred for the Pb UPD measurements at low scan rates. In all the subsequent experiments, Pb UPD measurements were performed in a solution with pH 3 and the composition HClO_4_ + NaClO_4_ +1 mM NaCl + 10 mM Pb(ClO_4_)_2_, at a total ClO_4_^−^ concentration equal to 0.1 M.

#### 3.2.3. Cyclic Voltammetry

**Figure 5:**
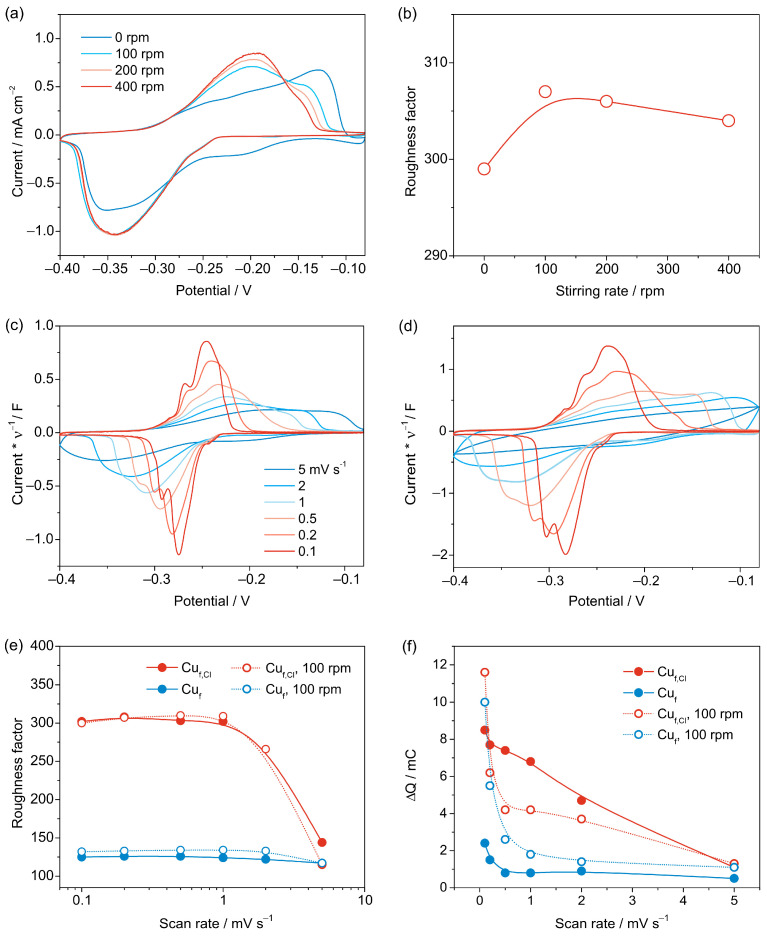
CVs of Cu_f,Cl_ sample in Pb UPD solution with and without magnetic stirring at different rates (**a**). The dependence of R_f_ on the stirring rate (**b**). CVs of Cu_f_ (**c**) and Cu_f,Cl_ (**d**) samples at different scan rates with stirring. Currents are normalized to the scan rate. R_f_ vs. scan rate with and without magnetic stirring (**e**). The difference between the cathodic and anodic charges vs. scan rate for the Cu_f_ and Cu_f,Cl_ samples (**f**).

In the case of porous materials, diffusion limitations may arise when the concentration of Pb^2+^ in solution is low. However, stirring the solution can alleviate this issue. We examined the impact of magnetic stirring on the voltammogram shape and R_f_ value. Figure 5a shows CVs of a Cu_f,Cl_ sample with and without magnetic stirring at different rates. Without stirring, the UPD peaks become a lot broader, but this does not affect the accuracy of total UPD charge estimation for the Cu_f,Cl_ sample with Rf ~300, as R_f_ values with and without stirring only differ by 3% (Figure 5b). However, for the samples with a much larger R_f_, stirring the solution would be crucial to obtain reliable estimates of the RSA from voltammetric measurements. Since subtraction of background currents and subsequent integration is usually easier for curves with narrow peaks, the solution was stirred at 100 rpm in further experiments.

While for compact copper deposits relatively high (10–20 mV·s^−1^) scan rates might be applied without losses of accuracy for R_f_ determination, CV measurements with high-surface-area deposits should be performed at a much lower scan rate, which depends on the R_f_ value. Figure 5c,d shows CVs of Cu_f_ and Cu_f,Cl_ samples, which demonstrate a 2.5-fold difference in the RSA. With an increase in the scan rate from 0.1 to 5 mV·s^−1^ for both samples, the shape of the curves changes dramatically. For the Cu_f,Cl_ sample, at 2 and 5 mV·s^−1^, no peaks can be observed in the CVs as ohmic-like distortions appear (Figure 5d). Correspondingly, the calculated R_f_ values for the Cu_f,Cl_ sample do not change appreciably when the curves are registered at 0.1–1 mV·s^−1^, yet sharply diminish when the scan rate reaches 2 mV·s^−1^ and further decays to 50% of its value at 5 mV·s^−1^ (Figure 5e). In contrast, for the Cu_f_ foam with a lower R_f_, only 11% of the RSA is lost when the scan rate changes from 0.1 to 1 mV·s^−1^. Notably, for the samples with R_f_ ~ 125 (Cu_f_) and 300 (Cu_f,Cl_), stirring has a minor effect on the Pb UPD charge (Figure 5e).

The last issue to be addressed concerns the influence of scan rate and stirring on the value of charge due to the background currents at cathodic potentials. Figure 5f shows the plots of the difference between the anodic and cathodic charges ΔQ vs. scan rate for the Cu_f_ and Cu_f,Cl_ samples with and without stirring. The charge due to the background currents is a lot higher for the high-surface-area Cu_f,Cl_ sample, which can be related to the larger size of the pores and hence higher diffusion-controlled currents for the oxygen reduction. Notably, the ΔQ values are much higher at low scan rates, e.g., 0.1 mV·s^−1^, which should be avoided to achieve higher accuracy in R_f_ determination. Stirring does not introduce any systematic shifts in the observed trends.

#### 3.2.4. Chronoamperometric Measurements

Practically, cyclic voltammetry is a more convenient method for UPD studies, as the qualitative-level information on the absence of problems related to reference electrode instability and ohmic distortions can be easily detected based on the shape of a CV. However, for highly dispersed porous deposits, low sweep rates are required, which makes CV measurements extremely time-consuming. In this case, chronoamperometric measurements should be preferred, as it was demonstrated for the UPD of Cu on porous gold [25,30].

**Figure 6:**
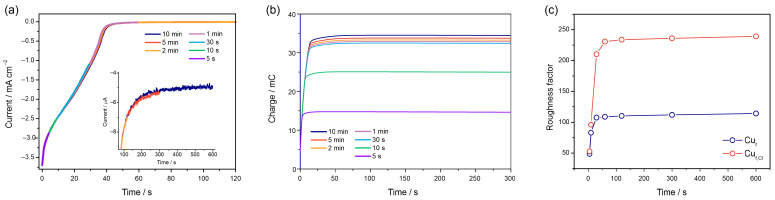
Current density transients of Pb UPD formation for 5, 10, 30 s and 1, 2, 5, 10 min on Cu_f,Cl_ at a potential −0.400 V (**a**) and subsequent Pb monolayer stripping charge at a potential of −0.080 V (**b**). Plot of R_f_ vs. time of Pb UPD formation (**c**).

To determine the UPD charge, Pb adatom deposition was performed potentiostatically at −0.400 V for Cu_f_ and Cu_f,Cl_ foams. Since residual oxygen reduction is the main process interfering with the Pb UPD [47], precise detection of the time required to complete the formation of UPD may become problematic. Figure 6a shows current transients registered during Pb monolayer formation at −0.4 V for 5 s, 10 s, 30 s, 1 min, 2 min, 5 min and 10 min. During the initial 30 s, the current transients exhibit a rapid decrease, reflecting the low charges at the start of Pb monolayer formation. This is followed by a region with a much lower slope, indicating the gradual filling of the Cu surface with Pb adatoms. Interestingly, UPD monolayer stripping takes place within the same time frame as UPD layer formation, unlike the situation observed in nanoporous gold, where stripping occurred much more quickly compared to deposition [30]. Pb monolayer formation is a relatively slow process, as background current values of ca. 5 µA·cm^−2^ can be attained only after 10 min of UPD formation at a constant potential, as shown in the inset of Figure 6a. For copper deposits with a higher surface area, longer UPD deposition time could be required. Figure 6b shows the dependence of charge required for the stripping of a Pb monolayer at the time of Pb UPD formation. Notably, the stripping charges demonstrate minor variation with the deposition time for times exceeding 30 s. Correspondingly, R_f_ values for Cu_f,Cl_ foams can be determined with the precision sufficient for most applications already after 1 min of Pb monolayer formation at −0.400 V (Figure 6c). For Cu_f_ samples, R_f_ changes are already minor after 30 s of deposition. The difference in the R_f_ values derived from low scan rate voltammetric and chronoamperometric measurements does not exceed 3%.

### 3.3. Reproducibility

**Figure 7:**
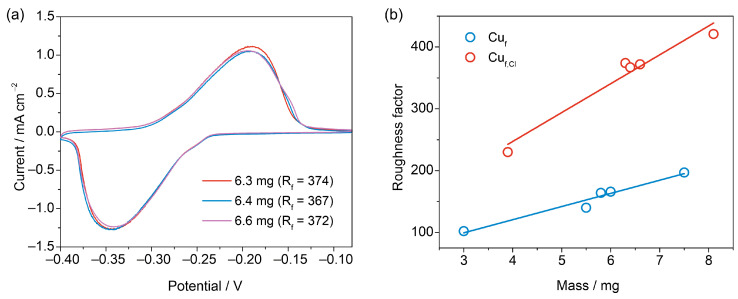
CVs of three Cu_f,Cl_ samples (3A, 30 s) in Pb UPD solution at 1 mV s^−1^ with stirring. The values of mass and R_f_ are indicated in the plot (**a**). Plot of R_f_ vs. mass for Cu_f_ and Cu_f,Cl_ samples (**b**).

The procedure for the determination of the RSA of copper foams allows checking the reproducibility of the R_f_ values. Figure 7a shows the CVs of three foams electrodeposited under identical conditions at 3 A for 20 s. The CVs in Pb UPD solutions exhibit merely identical profiles, which result in R_f_ values that differ by less than 3%. A linear correlation can be found between the R_f_ values and the masses of the Cu_f,Cl_ and Cu_f_ samples (Cu_f_ and Cu_f,Cl_ were deposited for 10, 20, and 30 s at 3 A and their masses were determined by weighting samples). Such plots (Figure 7b) can be used for calibration purposes for estimating the RSA values of highly dispersed Cu electrodeposits produced by the same method and having different masses.

Specific surface areas of the copper foams amount to 3.4 and 5.9 m^2^·g^−1^ for Cu_f_ and Cu_f,Cl_ samples with 10 s deposition time and do not depend significantly on the foam thickness. The specific surface area values are close to the specific surface areas obtained for similar copper foams using the BET method (~4 m^2^·g^−1^) [49]. This demonstrates that under the chosen experimental conditions, diffusion limitations in the pores of the copper deposits are efficiently minimized, and the whole surface area is accessible for the UPD process, which is an indication of the reliability of the RSA estimates derived from the Pb UPD measurements.

## 4. Conclusions

In this study, we obtained the RSA values of electrodeposited copper foams with different thicknesses using an optimized procedure for Pb UPD measurements. For copper foams deposited from acidic electrolytes with a Cl^−^ additive, roughness factors of ~400 could be achieved for 75 µm thick foams. For the foams deposited from a chloride-free solution, the maximal surface roughness is twice as low.

We have found that several parameters need to be controlled in order to obtain reliable estimates of surface roughness. The concentration of Pb^2+^ in solution should be in the tens of mM range when studying micro- or nanoporous deposits to minimize concentration polarization within the porous electrode. Stirring the solution enhances the diffusion of Pb^2+^, which is crucial for porous samples with a high surface area. The appropriate scan rate for RSA measurements by Pb UPD depends on the roughness factor. For samples with R_f_ between 100 and 400, scan rates of 0.5–1 mV·s^−1^ provide reliable RSA estimates, while higher scan rates can lead to underestimation of RSA. However, very low scan rates should be avoided as the coulometric balance may become violated due to the contribution of background processes, leading to errors in roughness factor calculations. For highly dispersed deposits, chronoamperometric UPD measurement protocols should be preferred. To ensure the reliability of the Pb UPD measurements, highly dispersed copper-based materials should not be kept in the UPD solution at open circuit potential or cycled for extended periods as this can cause a decrease in surface area. The reproducibility of R_f_ values for copper foams is quite high, with differences between samples obtained using the same method and having similar masses not exceeding 3%.

We believe that the optimized conditions for Pb UPD measurements in the RSA determination of high-surface-area copper-based materials will be valuable for characterizing electrodes for electrocatalytic applications. This method is preferred over approaches based on double-layer capacitance measurements or calibration curves constructed from surface oxidation peaks due to its higher accuracy and minimal interference from background processes.

## Data Availability

The data presented in this study are available on request from the corresponding author.

## Supplementary Materials

The following supporting information can be downloaded at: https://www.mdpi.com/article/10.3390/nano13233011/s1, Section S1: Current efficiency and total pore volume determination; Figure S1: SEM images of the Cu_f_ (left) and Cu_f,Cl_ (right) specimens; Figure S2: Bright-field STEM images of Cu_f_ (A) and Cu_f,Cl_ (B) particles; Figure S3: CV of a copper electrode (electropolished foil) in 0.1 M HClO_4_ + 1 mM Pb(ClO_4_)_2_ and 0.1 M HClO_4_ + 1 mM Pb(ClO_4_)_2_ + 1 mM HCl solutions at a potential scan rate of 10 mV s^−1^.
