# Supplementary material for: Roughness Factors of Electrodeposited Nanostructured Copper Foams
Source: Nanomaterials (Basel). 2023 Nov 23;13(23):3011. doi: 10.3390/nano13233011 (PMC10708413; doi:10.3390/nano13233011)
Supplement: Supplementary file 1 [file nanomaterials-13-03011-s001.zip › nanomaterials-2714582-supplementary.pdf]

Supplementary Information

Roughness Factors of Electrodeposited Nanostructured Copper Foams

Eduard E. Levin [1,2,*], Dmitriy A. Morozov [1,3], Vsevolod V. Frolov [1], Natalia A. Arkharova [2], Dmitry N. Khmelenin [2], Evgeny V. Antipov [1,3] and Victoria A. Nikitina [1,3,*]

1 Department of Chemistry, Lomonosov Moscow State University, Moscow 119991, Russia; dmitrii.morozov@chemistry.msu.ru (D.A.M.); frolov_vsevolod@mail.ru (V.V.F.); antipov@inorg348-1.chem.msu.ru (E.V.A.)
2 Federal Scientific Research Centre "Crystallography and Photonics" of the Russian Academy of Sciences, Moscow 119333, Russia; natalya.arkharova@yandex.ru (N.A.A.); xorrunn@gmail.com (D.N.K.)
3 Center for Energy Science and Technology, Skolkovo Institute of Science and Technology, Moscow 121205, Russia
* Correspondence: levin@elch.chem.msu.ru (E.E.L.); v.nikitina@skoltech.ru (V.A.N.)

S1. Current Efficiency and Total Pore Volume Determination

The current efficiency was determined by weighing with calculation of absolute errors. The total mass *M* of the electrodeposited copper, taking into account partial oxidation to Cu_2O, was determined as:

$$M = m * w_1 + \frac{2 * M(Cu)}{M(Cu_2O)} * w_2 * m,$$

where *m* is the weighted mass, w_1 is the weight fraction of *Cu*, determined by XRD, w_2 is the weight fraction of Cu_2O, determined by XRD, *M*(*Cu*) and $M(Cu_2O)$ are molecular weights of copper and cupric oxide, respectively.

Current efficiency, *CE*, is:

$$CE = \frac{M}{m_F},$$

where m_F is the mass of Cu calculated using Faraday's law.

Total pore volume P_v is determined as:

$$P_v = 1 - \frac{M}{S * H * \rho},$$

where *S* is the support surface area determined using image analysis, *H* is the average height of the deposited foam determined from its cross-section images and ρ is the *Cu* density (8.96 g cm^{-3}).

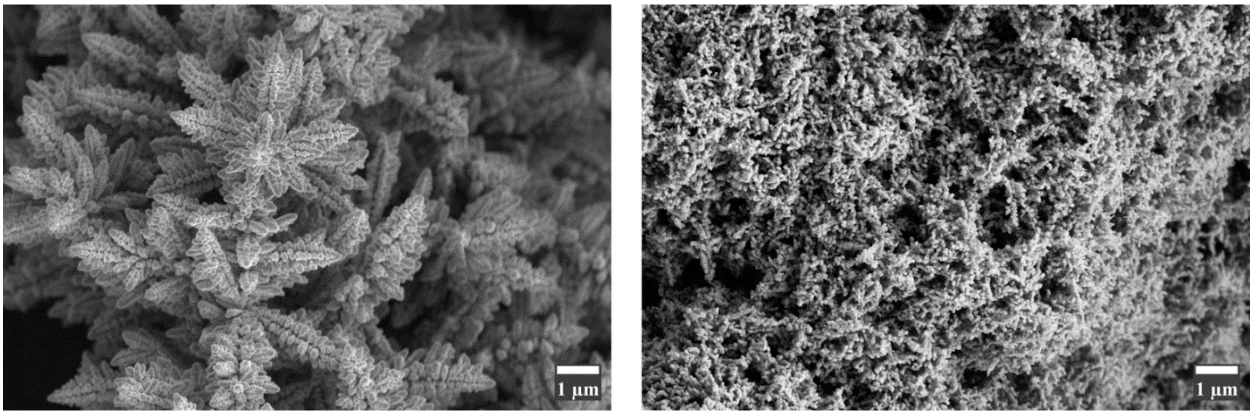

Figure S1. SEM images of the Cu_f (left) and $Cu_{f,Cl}$ (right) specimens.

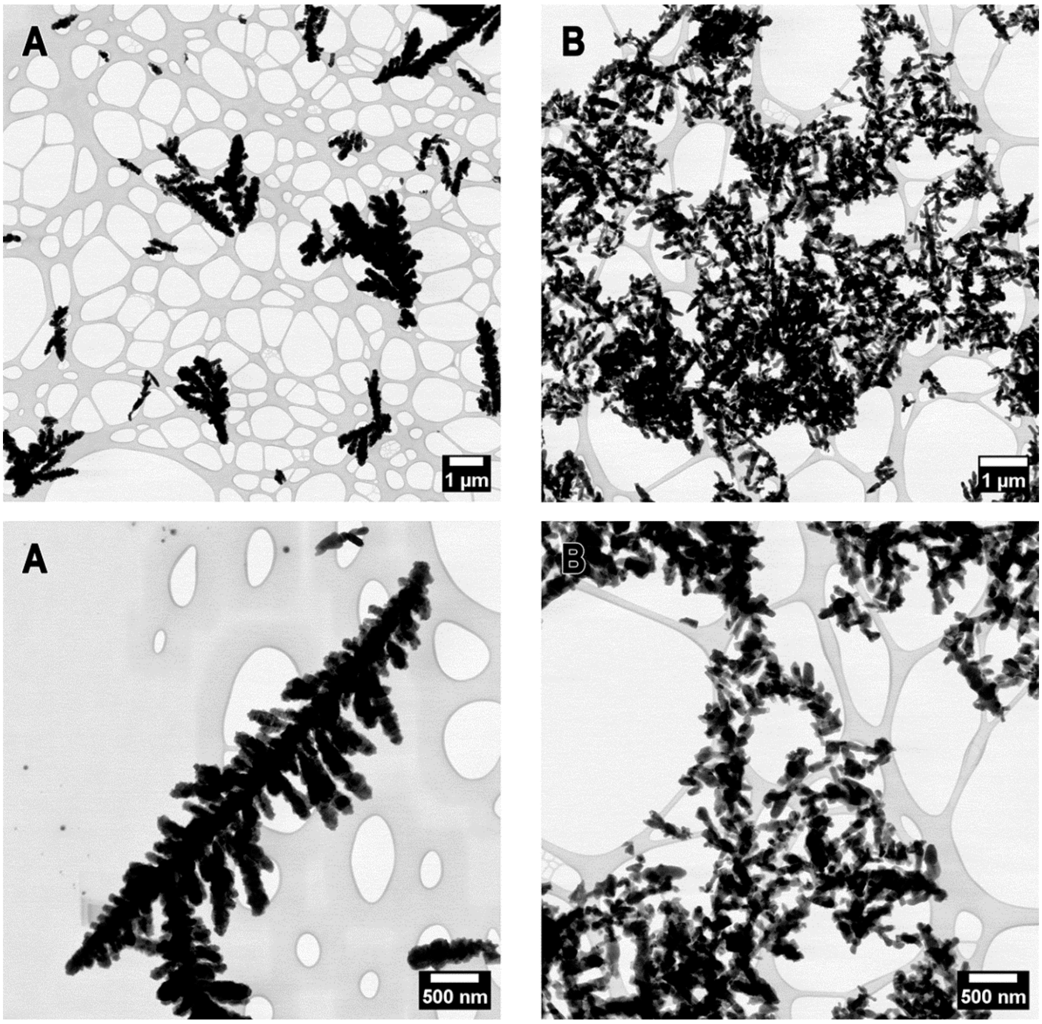

Figure S2. Bright-field STEM images of Cu_f (A) and $Cu_{f,Cl}$ (B) particles.

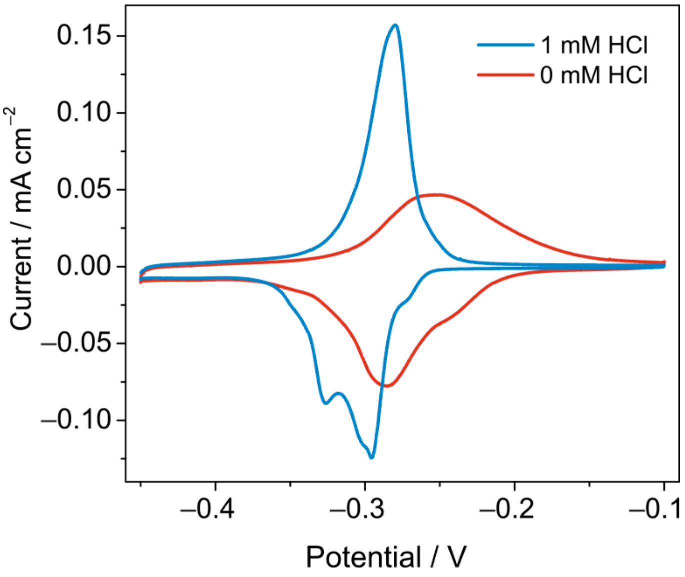

Figure S3. CV of a copper electrode (electropolished foil) in 0.1 M $HClO_4$ + 1 mM $Pb(ClO_4)_2$ and 0.1 M $HClO_4$ + 1 mM $Pb(ClO_4)_2$ + 1 mM HCl solutions at a potential scan rate of 10 mV s^{-1}.
